# 3D-QSAR Studies on Thiazolidin-4-one S1P_1_ Receptor Agonists by CoMFA and CoMSIA

**DOI:** 10.3390/ijms12106502

**Published:** 2011-09-28

**Authors:** Chuiwen Qian, Junxia Zheng, Gaokeng Xiao, Jialiang Guo, Zhaoqi Yang, Li Huang, Wei Chao, Longyi Rao, Pinghua Sun

**Affiliations:** 1 College of Biology Science and Technology, Jinan University, Guangzhou 510632, China; E-Mails: litmony@sohu.com (C.Q.); thuangli@jnu.edu.cn (L.H.); 2 Faculty of Chemical Engineering and Light Industry, Guangdong University of Technology, Guangzhou 510006, China; E-Mail: junxiazheng@163.com; 3 Guangdong Province Key Laboratory of Pharmacodynamic Constituents of TCM and New Drugs Research, College of Pharmacy, Jinan University, Guangzhou 510632, China; E-Mails: gkxiao@yahoo.com.cn (G.X.); janalguo@126.com (J.G.); pharmacydoctor@163.com (Z.Y.); 271362773@qq.com (W.C.); longyi_ke@163.com (L.R.)

**Keywords:** CoMFA, CoMSIA, QSAR, thiazolidin-4-one, S1P_1_ receptor agonists

## Abstract

Selective S1P_1_ receptor agonists have therapeutic potential to treat a variety of immune-mediated diseases. A series of 2-imino-thiazolidin-4-one derivatives displaying potent S1P_1_ receptor agonistic activity were selected to establish 3D-QSAR models using CoMFA and CoMSIA methods. Internal and external cross-validation techniques were investigated as well as some measures including region focusing, progressive scrambling, bootstraping and leave-group-out. The satisfactory CoMFA model predicted a *q*^2^ value of 0.751 and an *r*^2^ value of 0.973, indicating that electrostatic and steric properties play a significant role in potency. The best CoMSIA model, based on a combination of steric, electrostatic, hydrophobic and H-bond donor descriptors, predicted a *q*^2^ value of 0.739 and an *r*^2^ value of 0.923. The models were graphically interpreted using contour plots which gave more insight into the structural requirements for increasing the activity of a compound, providing a solid basis for future rational design of more active S1P_1_ receptor agonists.

## 1. Introduction

Sphingosine-1-phosphate (S1P), a widespread lysophospholipid, binds five specific G-protein coupled receptors (S1P_1_-S1P_5_) [1–4] and exerts a variety of biological activities such as vascular maturation, cell survival, proliferation, differentitation, migration and chemotaxis [5–7]. The S1P_1_ receptor only couples to Gαi/o while the other S1P receptors are promiscuous with respect to G-protein coupling, activating G_12/13_ in addition to Gαi/o and also Gαq in the case of S1P_2_ and S1P_3_ [8]. S1P_1_ modulates egress of T-lymphocytes from thymus and peripheral lymphoid organs [9], and experiments [10,11] have demonstrated that targeting S1P_1_ is sufficient to cause lymphocyte sequestration to thymus and lymphoid organs, without affecting the innate immune system and even cellular reactivity of lymphocytes to antigen challenge. On the other hand, activation of the S1P_3_ does not relate to lymphocyte recirculation but links to some undesirable effects such as bronchoconstriction, heart rate reduction and pulmonary epithelial leakage [12–15]. Selective S1P_1_ receptor agonists are promisingly developed as a novel immunomodulator. S1P_1_ receptor has been considered a potential target for a variety of immune-mediated diseases, including rheumatoid arthritis, psoriasis and multiple sclerosis disease. Recently, a novel class of S1P_1_ receptor agonists based on the 2-imino-thiazolidin-4-one scaffold has been reported [8] and ligand-based drug design techniques will be employed to guide the synthesis of future generations of S1P_1_ receptor agonists since the crystal structure of S1P_1_ receptor remained unclear.

Three-dimensional quantitative structure-activity relationship (3D-QSAR) techniques are useful methods of ligand-based drug design by correlating physicochemical descriptors from a set of related compounds to their known molecular activity or molecular property values [16,17]. Many researchers [18,19] have carried out quantitative structure activity relationship (QSAR) studies on thiazolidin-4-ones as anti-HIV agents, but the present work reports the first application of QSAR to study thiazolidin-4-ones as potent S1P1 receptor agonists [8]. We studied 61 2-imino-thiazolidin-4-one derivatives using comparative molecular field analysis (CoMFA) [20] and comparative molecular similarity indices analysis (CoMSIA) [21]. The satisfactory QSAR models obtained provide a solid basis for future rational design of more active and selective S1P_1_ receptor agonists within the family of S1P receptors.

## 2. Results and Discussion

### 2.1. CoMFA Analysis

**Figure 1:**
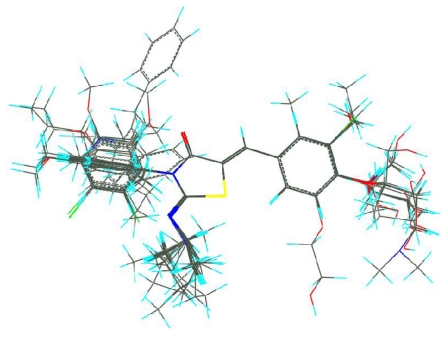
Molecular alignment of 2-imino-thiazolidin-4-one derivatives.

**Figure 2:**
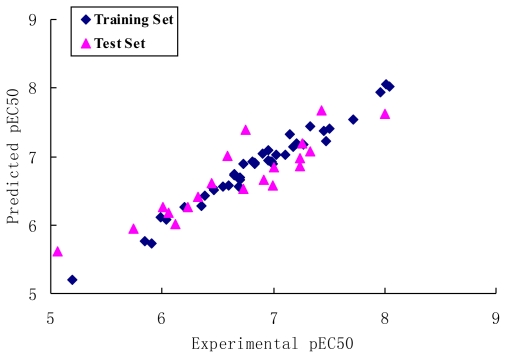
Graph of experimental *versus* predicted pEC50 of the training set and the test set using the CoMFA model.

**Figure 3:**
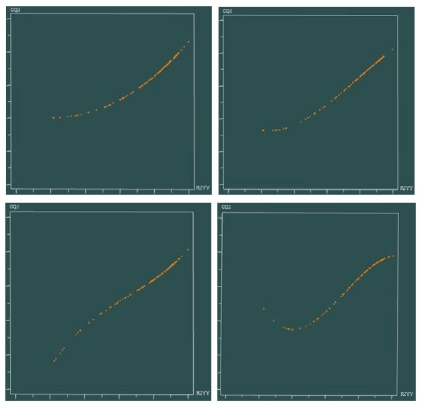
Variation fitted curves for progressive scrambling analyses with random number seed: (**left upper**) 3 components; (**right upper**) 4 components; (**left lower**) 5 components; (**right lower**) 6 components.

The most active molecule **60** was selected as the template for alignment (Figure 1) and the CoMFA model provided a cross-validation *q*^2^ value of 0.751 with 5 components and an *r*^2^ value of 0.973 (Table 1). The activity values predicted for the tested compounds are in good agreement with the experimental values (Figure 2) and the *r*^2^ _pred_ value of 0.904 further confirms that the model is reliable and accurate with higher predictive capacity. The steric and electrostatic field contributions to the final model were 54.5% and 45.5%, respectively. The results of the progressive scrambling showed that the value for the slope in the 5 component model is acceptable (Figure 3), and the optimum statistics are also seen for 5 components because cSDEP is at a minimum with *Q*^2^ at a maximum (Table 2).

### 2.2. CoMSIA Analysis

**Figure 4:**
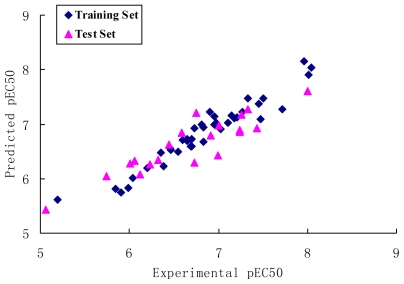
Graph of experimental *versus* predicted pEC50 of the training set and the test set using the best CoMSIA model 6.

Eight CoMSIA models were generated using combinations of two, three, four, and all five descriptors as shown in Table 3. Model 6, based on four descriptors of steric, electrostatic, hydrophobic and H-bond donor fields was found to be the most accurate yielding a *q*^2^ value of 0.739 and an *r*^2^ value of 0.923. The Group cross *q*^2^ value of 0.740, bootstrapped value of 0.973 ± 0.007 and test set *r*^2^ value of 0.730 further approve that it is the best CoMSIA model. The predicted values are consistent with the experimental data (Figure 4). The steric field explains 12.5% of the variances, the electrostatic field explains 29.0%, the hydrophobic field for 30.3% and the hydrogen-bond donor for 28.2% of the variance.

### 2.3. CoMFA Contour Maps

**Figure 5:**
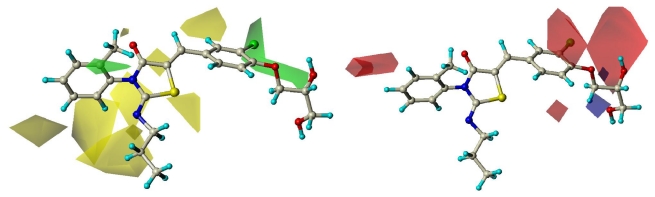
CoMFA field contour maps for active compound **60**. Steric fields (**Left**): Green fields indicate steric bulk favored, yellow fields indicate steric bulk disfavored. Electrostatic fields (**Right**): Blue fields indicate electropositive groups favored, red fields indicate electronegative groups favored.

The results of 3D-QSAR models are presented in the contour coefficient maps as shown in Figure 5. The CoMFA steric contour map of the most active compound **60** shows a large green polyhedron around 3, 4-positions of 5-benzylidene and a small green one locates near 2-position of N_3_-phenyl ring, suggesting that bulky substituents are preferred in these regions. Large yellow polyhedra at 2-position of 5-benzylidene and around 2-substituent at the iminothiazolidinone scaffold indicate that bulks are disfavored here. These may be the reason why compound **60** with a chlorine atom and a hydroxylethyloxyl substitution at 3- and 4-positions of 5-benzylidene respectively, a methyl group linking to 2-position of 3-phenyl ring substituted at iminothiazolidinone ring, and no substitutions at 2-position of 5-benzylidene ring, showed the most active. Similar to compound **60**, compounds **58**, **59**, **61** displayed almost identical potency. On the other hand**,** compound **56,** with the reverse of substitution at each corresponding position, showed very low activity. The contour maps also show that compounds **18**, **25–27**, **40** exhibited much higher potency than **19**, **30–32**, **46** correspondingly. At the same time, the steric contour illustrated a weak trend for a reducing chain length to steadily improve the compound’s potency. For example, the activity of compounds **12**, **11**, **10**, **9** was decreased in turn.

For the electrostatic contour plots, two small blue polyhedra and a large red polyhedron are both located around the side chain linking to 4-position of 5-benzylidene, indicating that negatively charged groups and electron donating groups are both available in these regions. A large red polyhedron located at 3-position of 5- benzylidene and around 3, 4-positions of N_3_-phenyl ring, indicating that electron-withdrawing groups are preferred at these positions. That is why compounds **26–28** were shown to be more potent than compounds **29–32** with electron-donating groups, and compounds **50–54** without any substituents at 3-position of 5-benzylidene displayed more potency than the corresponding compound **56**. Combined with its steric contour map, the chloro-substituent at 3-position of the benzylidene ring is essential group for compound’s potency on S1P_1_.

### 2.4. CoMSIA Contour Maps

**Figure 6:**
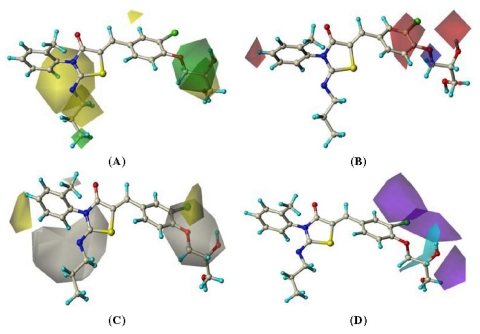
CoMSIA fields. The CoMSIA fields from model 6 are shown with active compound 60. (**A**) Steric fields, green indicates steric bulk favored, yellow indicates bulk disfavored; (**B**) Electrostatic fields, blue indicates electropositive groups favored, red fields indicate electronegative groups favored; (**C**) Hydrophobic fields, yellow indicates favored, gray disfavored; (**D**) H-bond donor field, cyan indicates donor favored, purple disfavored.

The best CoMSIA model contour maps of the most active analog (compound **60** in Table 4) are shown in Figure 6. Its steric and electrostatic contour plots (Figure 6A, B) correlate well with the CoMFA contour maps described above. Figure 6C displays the hydrophobic contour map represented by yellow and white polyhedra. A large yellow polyhedron and a small polyhedron located around 2, 3, 4-positions of N_3_-phenyl ring respectively, indicating that hydrophobic substituents on ligands in these regions can be favored and the white ones disfavored such substituents. That is why compounds **58**–**61** with a methyl group at the region showed far more potent than others. Figure 6D shows the CoMSIA hydrogen-bond donor fields denoted by cyan and purple contours. Cyan contours represent regions where hydrogen-bond donor substituents are preferred and purple contours indicate unfavorable regions. One large cyan contour around the side chain linking to 4-position of 5-benzylidene indicate that the free OH at 4-position or on its side chain is necessary for potencies. A large purple contour at 2, 3-positions respectively shows where such substituents may be disfavored. That is why compound **40** showed more potent than **41** while compound **56** was less active than compounds **50–54** and **57**.

## 3. Materials and Methods

### 3.1. Data Set

Sixty-one compounds in the present study were taken from the published works of Martin H. Bolli and co-workers [8], and some molecules whose activity was shown not to be very exact from the experimental study were excluded. The structures of the molecules and their biological data are given in Table 4. For convenience, the EC50 values of S1P_1_ receptor agonists were often converted to their negative logarithm (pEC50) values. The pEC50 values of these compounds have a span of 3.0 log units from 5.06 to 8.04, providing a broad and homogenous data set for 3D-QSAR study [22]. 20 compounds were randomly selected as the test set based on the structural diversity while the remaining 41 compounds were taken as the training set.

Three-dimensional structure building and all modeling were performed using the SYBYL 8.1 program package of Tripos, Inc. 3D structures of all compounds were constructed using the Sketch Molecule module. Structural energy minimization was carried out using the standard Tripos molecular mechanics force field and Gasteiger–Hückle charge, with the convergence criterion set at 0.05 kcal/(Å mol) and the max iterations for the minimization set to 2000.

One method of 3D-QSAR optimization is known as region focusing [23]. However, application of region focusing in this study resulted in a little decrease from 0.751 to 0.739 for the internal validity and from 0.973 to 0.958 for the non-validated *r^2^*. Obviously, the region focusing is not essential in this CoMFA model.

### 3.2. Molecular Alignment

A good alignment is the most important element for CoMFA and CoMSIA analysis although probe atom type, lattice shifting step size and over all orientation of the aligned compounds may have a bearing on their results [24]. The quality and the predictive ability of the model are directly dependent on the alignment rules. Once the active conformation was determined by energy minimization using Powell method and Tripos standard force field [25] with a distance-dependent dielectric function, the alignment was performed based on some rules such as substructure overlap, pharmacophore overlap and docking [26]. In order to produce a more robust CoMFA and CoMSIA models with a good cross-validated *r^2^* value, the 5-benzylidenylthizaolindin-4-one with structural rigidity was selected as the common substructure to overlap and to align all of the molecules and the most active compound **65** was used as the alignment template. Alignment of all compounds was shown in Figure 1. It can be seen that all the compounds studied have similar active conformations.

### 3.3. Partial Least Squares (PLS) Analysis

PLS analysis [27], used to linearly correlate the 3D-QSAR fields to biological activity values, was first carried out by the leave-one-out (LOO) and leave-group-out (10 compound groups) cross-validation methods, respectively, to determine cross-validated *r*^2^ (*q*^2^) values and the optimal number of components, in which one compound was removed from the data set and its activity was predicted using the model from the rest of the data set. Non-cross-validation was then performed to establish the final 3D-QSAR model using the optimal number of components, in which conventional correlation coefficient (*r*^2^), standard errors of estimate (SEE), and *F* ratio between the variances of calculated and observed activities were given.

The optimal number of components, usually corresponding to the highest cross-validated squared coefficient (*q*^2^), was selected on the basis of the lowest standard error of prediction (SEP) and avoiding over-fitting the models. A higher component was accepted and used only when the *q*^2^ differences between two components was larger than 10%. The *q*^2^ has been a good indicator of the accuracy of actual predictions. In general, *q*^2^ values can be separated into three categories: *q*^2^ > 0.6 means a fairly good model, *q*^2^ = 0.4 – 0.6 means a questionable model, and *q*^2^ < 0.4 a poor model [27]. To further assess the robustness of the derived models, bootstraping analysis (10 runs) was also utilized to calculate confidence intervals for the *r*^2^ and SEE [28,29].

### 3.4. CoMFA Studies

Three-dimensional grid spacing was set at 2 Å in the *x*, *y*, and *z* directions and a 3D cubic lattice consisted its grid region that extended at least 4 Å beyond van der Waals volume of all aligned molecules in all directions. Steric energy (Lennard-Jones potential) and electrostatic energy (Coulomb potential) were calculated using the Tripos force field for each molecule, and the sp^3^-hybrized carbon atom with a +1 charge was taken as the probe atom to determine the magnitude of the field values. All energies that exceeded the cutoff value of 30 kcal/mol were ignored for the reduction of domination by large steric and electrostatic energies. With standard options for scaling of variables, the regression analysis was carried out using partial least squares (PLS) method [27]. To improve the signal to noise ratio, the column filtering was set to 2.0 kcal/mol, then those lattice points whose energy variation was below this threshold were automatically omitted [30]. The final model was developed with the optimum number of components to yield a non cross-validated *r*^2^ value. Despite being unable to describe all of the binding forces, CoMFA is still a widely useful tool for QSAR analysis at 3D level.

### 3.5. CoMSIA Studies

CoMSIA is similar to CoMFA based on the same assumption that changes in binding affinities of ligands are related to changes in molecular properties represented by fields. Moreover, for CoMSIA, besides steric and electrostatic fields, three other different fields (hydrophobic, hydrogen bond donor, and hydrogen bond acceptor) are calculated [31]. A Gaussian function was introduced to determine the distance between the probe atom and the molecule atoms. Similarity indices inside and outside different molecular surfaces can be calculated at all grid points, however only outside indices were calculated in CoMFA. Equation used to calculate the similarity indices is as follows:

$$A_{F,K(j)}^{q}=\sum_{i}W_{probe,k}W_{ik}e^{-\alpha r_{iq}^{2}}$$

Where, *A* is the similarity index at grid point *q*, summed over all atoms *i* of the molecule *j* under investigation. *W*_probe, k_ is the probe atom with radius 1 Å, charge +1, hydrophobicity +1, hydrogen bond donating +1 and hydrogen bond accepting +1. *W*_ik_ is the actual value of the physicochemical property *k* of atom *i. r*_iq_ is the mutual distance between the probe atom at grid point *q* and atom *i* of the test molecule. α is the attenuation factor whose optimal value is normally between 0.2 and 0.4, with a default value of 0.3 [32,33].

### 3.6. Sensitivity of a PLS Model

Most members of the data set, especially for large data sets, may have “twins” which make a near twin of each left-out molecule likely remain in the training data and usually obtain good predictions, so the *q*^2^ statistic obtained from cross-validation may give a false sense of confidence [34]. Progressive scrambling is used to test the model’s stability by determining the sensitivity of a QSAR model to small systematic perturbations of the response variable [35,36]. The values of *Q*^2^, cSDEP, and d*q*^2^/d*r*^2^ _yy′_ are used to interpret the predictivity of the model without the potentially confusing redundancy, in which the *Q*^2^ statistic is an estimate of the predictivity of the model after removing the effects of redundancy, and the cSDEP statistic is an estimated cross-validated standard error at a specific critical point for *r*^2^ _yy′_, and d*q*^2^/d*r*^2^ _yy′_ means slope of *q*^2^ evaluated at the specified critical point with respect to *r*^2^ _yy′_.

$$cSDEP[r_{{yy}^{\prime }}^{2}]=a_{0}+a_{1}(r_{{yy}^{\prime }}^{2})+a_{2}{\left(r_{{yy}^{\prime }}^{2}\right)}^{2}+a_{3}{\left(r_{{yy}^{\prime }}^{2}\right)}^{3}$$

$$q^{2}(r_{{yy}^{\prime }}^{2})=b_{0}+b_{1}(r_{{yy}^{\prime }}^{2})+b_{2}{\left(r_{{yy}^{\prime }}^{2}\right)}^{2}+b_{3}{\left(r_{{yy}^{\prime }}^{2}\right)}^{3}$$

$$\frac{{dq}^{2}}{{dr}_{{yy}^{\prime }}^{2}}=b_{1}+2b_{2}(r_{{yy}^{\prime }}^{2})+3b_{3}{\left(r_{{yy}^{\prime }}^{2}\right)}^{2}$$

Here, y_′_ indicates the perturbed (scrambled) responses and the statistic *r*^2^ _yy′_ expresses the degree of correlation between the perturbed responses and the original ones. The *Q*^2^ statistic obtained in this way is very conservative, in that it is necessarily reduced by the level of noise introduced to remove redundancy, so a value as low as 0.35 will signify that the original, unperturbed model is robust [33]. The d*q*^2^/d*r*^2^ _yy′_ means what extent the model changes with small changes to the dependent variable. In a stable model, changing proportionally with small changes in underlying data, has a slope near unity while unstable models change greatly with small changes in underlying response values and its effective slope is generally greater than 1.2. This method was employed to verify the optimal number of components and test the cross-validation against the possibility of such a redundancy in our training set [37].

### 3.7. Predictive Correlation Coefficient

*q*^2^ is a useful but not sufficient criterion for model validation, so an external test set (*r*^2^ _pred_) [38] was claimed for the estimation of predictive ability. Equation of predictive values *r*^2^ _pred_ is as follows:

$$r_{pred}^{2}=1-(PRESS/SD)$$

Therein, SD means the sum of squared differences between the measured activities of the test set and the average measured activity of the training set.

## 4. Conclusions

Although many researchers have carried out quantitative structure activity relationship (QSAR) studies on thiazolidin-4-one as anti-HIV agents, the present work reports the first application of QSAR to study thiazolidin-4-ones as potent S1P1 receptor agonists [8]. We studied 61 2-imino-thiazolidin-4-one derivatives using CoMFA and CoMSIA, and some predictive 3D-QSAR models have been developed. Both CoMFA and CoMSIA models provided good statistical results in terms of *q*^2^ and *r*^2^ values, suggesting the significant correlations of molecular structures with its biological activities. Compared with CoMFA, CoMSIA provided a slightly better statistical model. The final CoMSIA model was generated from steric, electrostatic, hydrophobic and hydrogen-bond donor fields and validated by a variety of methods including crossvalidation, non-crossvalidation and test set predictions. The model developed has high internal validity (*q*^2^ above 0.6) and high predictive ability (test set *r*^2^ above 0.7). Compared with SAR summarized by Martin H. Bolli, the results of 3D-QSAR models presented in the contour coefficient maps further revealed how steric, electrostatic, hydrophobic and hydrogen-bond donor modifications should significantly affect the bioactivities of these compounds. Thus, the results of the quantitative structure activity relationships (QSAR) studies gave an insight to design new S1P_1_ receptor agonists which can effectively treat a variety of immune-mediated diseases.

## Figures and Tables

**Table 1:** Statistical results of CoMFA and the best CoMSIA models.

	CoMFA	CoMSIA (Model 6)
***PLS*statistics**		
LOO cross *q*^2^/SEP	0.751/0.320	0.739/0.328
Group cross *q*^2^/SEP	0.744/0.325	0.740/0.332
Non-validated *r*^2^/SEE	0.973/0.106	0.923/0.178
F	250.674	84.398
*r*^2^_bootstrap_	0.985 ± 0.006	0.973 ± 0.007
*S*_bootstrap_	0.074 ± 0.041	0.115 ± 0.064
Optimal components	5	5
**Field distribution%**		
Steric	54.5	12.5
Electrostatic	45.5	29.0
Hydrophobic		30.3
H-bond donate		28.2
*r*^2^_pred_	0.904	0.730

**Table 2:** Progressive scrambling results of the CoMFA model.

Components	*Q^2^*	cSDEP	d*q^2^*/d*r^2^*_yy′_
3	0.403	0.531	1.391
4	0.350	0.585	1.230
5	0.540	0.542	1.021
6	0.393	0.517	1.427
7	0.433	0.583	1.391

**Table 3:** Results of CoMSIA models using combinations of the four field descriptors.

Model	Descriptors	LOO cross *q*^2^/SEP	Group cross *q*^2^/SEP	Bootstrap *r*^2^	Bootstrapped SEE	Non-validated *r*^2^/SEE
1	S and E	0.630/0.396	0.608/0.408	0.953 ± 0.011	0.129 ± 0.070	0.945/0.153
2	S, E and H	0.735/0.310	0.723/0.324	0.873 ± 0.036	0.213 ± 0.127	0.871/0.221
3	S, E and A	0.637/0.386	0.654/0.377	0.942 ± 0.017	0.152 ± 0.084	0.921/0.181
4	S, E and D	0.695/0.359	0.692/0.361	0.952 ± 0.020	0.143 ± 0.090	0.929/0.173
5	S, E, D and A	0.657/0.381	0.658/0.381	0.930 ± 0.021	0.175 ± 0.088	0.918/0.187
6	S, E, D and H	0.739/0.328	0.740/0.332	0.973 ± 0.007	0.115 ± 0.064	0.923/0.178
7	S, E, A and H	0.726/0.334	0.731/0.334	0.960 ± 0.011	0.139 ± 0.070	0.920/0.185
8	S, E, D, A and H	0.719/0.340	0.709/0.351	0.953 ± 0.018	0.142 ± 0.077	0.910/0.192

**Table 4:** 2-imino-thiazolidin-4-one derivatives and their experimental and predicted agonistic activity.

No	O									

	R_1_	R_2_	R_3_	R_4_	R_5_	Exp.	CoMFA		CoMSIA	

							Pred.	Res.	Pred.	Res.
1	dimethyl-mino	phenyl	H	Cl	OH	7.208	7.188	0.020	7.129	0.079
2 *	methyl	phenyl	H	Cl	OH	6.005	6.260	−0.255	6.280	−0.275
3	ethyl	phenyl	H	Cl	OH	6.730	6.884	−0.154	6.921	−0.191
4	n-propyl	phenyl	H	Cl	OH	7.174	7.149	0.025	7.107	0.067
5	n-butyl	phenyl	H	Cl	OH	6.951	7.097	−0.146	7.143	−0.192
6 *	isopropyl	phenyl	H	Cl	OH	7.328	7.078	0.250	7.267	0.061
7	sec-butyl	phenyl	H	Cl	OH	6.992	6.900	0.092	6.951	0.041
8	tert-butyl	phenyl	H	Cl	OH	6.833	6.887	−0.054	6.939	−0.106
9	cyclopropyl	phenyl	H	Cl	OH	6.987	6.921	0.066	6.998	−0.011
10	cyclobutyl	phenyl	H	Cl	OH	6.695	6.558	0.137	6.601	0.094
11	cyclopentyl	phenyl	H	Cl	OH	6.460	6.548	−0.088	6.564	−0.104
12 *	cyclohexyl	phenyl	H	Cl	OH	6.234	6.265	−0.031	6.260	−0.026
13 *	isopropyl	isopropyl	H	Cl	OH	7.237	6.865	0.372	6.890	0.347
14	isopropyl	n-hexyl	H	Cl	OH	6.550	6.555	−0.005	6.495	0.010
15 *	isopropyl	cyclohexyl	H	Cl	OH	8.000	7.624	0.376	7.610	0.390
16 *	isopropyl	ethoxycarbonylethyl	H	Cl	OH	6.320	6.405	−0.085	6.346	−0.026
17 *	isopropyl	allyl	H	Cl	OH	7.259	7.185	0.074	7.177	0.082
18	isopropyl	2-methylphenyl	H	Cl	OH	7.468	7.232	0.236	7.089	0.379
19	isopropyl	3-methylphenyl	H	Cl	OH	6.959	6.947	0.012	6.997	−0.038
20	isopropyl	4-methylphenyl	H	Cl	OH	7.108	7.021	0.087	7.025	0.083
21	isopropyl	2,6-dimethylphenyl	H	Cl	OH	6.813	6.929	−0.116	6.987	−0.174
22	isopropyl	2,3-dimethylphenyl	H	Cl	OH	7.143	7.321	−0.178	7.163	−0.020
23 *	isopropyl	2,4-dimethylphenyl	H	Cl	OH	6.747	7.394	−0.647	7.202	−0.455
24	isopropyl	2-ethylphenyl	H	Cl	OH	6.907	7.045	−0.138	7.226	−0.319
25	isopropyl	2-chlorophenyl	H	Cl	OH	7.268	7.178	0.090	7.218	0.050
26	isopropyl	3-chlorophenyl	H	Cl	OH	7.456	7.377	0.079	7.376	0.080
27	isopropyl	3-chloro-2-methylphenyl	H	Cl	OH	7.509	7.399	0.110	7.472	0.037
28	isopropyl	3-chloro-4-methylphenyl	H	Cl	OH	7.328	7.448	−0.120	7.481	−0.153
29	isopropyl	2-methoxylphenyl	H	Cl	OH	6.975	6.933	0.042	7.019	−0.044
30*	isopropyl	3-methoxylphenyl	H	Cl	OH	6.587	7.015	−0.428	6.839	−0.252
31	isopropyl	4-methoxylphenyl	H	Cl	OH	6.600	6.574	0.026	6.707	−0.107
32 *	isopropyl	2,4-dimethoxyphenyl	H	Cl	OH	6.058	6.182	−0.124	6.321	−0.263
33 *	isopropyl	3-pyridyl	H	Cl	OH	6.449	6.613	−0.164	6.623	−0.174
34	isopropyl	benzyl	H	Cl	OH	6.201	6.268	−0.067	6.195	0.006
35	isopropyl	phenethyl	H	Cl	OH	6.034	6.077	−0.043	6.013	0.021
36 *	isopropyl	4-phenylbutyl	H	Cl	OH	5.741	5.952	−0.211	6.053	−0.312
37	isopropyl	phenyl	H	H	H	5.908	5.726	0.182	5.753	0.155
38 *	isopropyl	phenyl	H	H	OH	6.914	6.653	0.261	6.794	0.120
39	isopropyl	phenyl	H	F	OH	6.648	6.719	−0.071	6.726	−0.078
40 *	isopropyl	phenyl	H	CH_3_	OH	7.432	7.674	−0.242	6.928	−0.496
41	isopropyl	phenyl	H	OCH_3_	OH	6.699	6.654	0.045	6.597	0.102
42	isopropyl	phenyl	H	H	OCH_3_	6.697	6.696	0.001	6.731	−0.034
43	isopropyl	phenyl	H	OCH_3_	OCH_3_	6.650	6.739	−0.089	6.695	−0.045
44	isopropyl	phenyl	H	H	NH(CH_3_)_2_	6.830	6.914	−0.084	6.669	0.161
45	isopropyl	phenyl	H	H	Br	5.984	6.112	−0.128	5.829	0.155
46 *	isopropyl	phenyl	H	OCH_3_	H	6.733	6.521	0.212	6.299	0.434
47 *	isopropyl	phenyl	H	OH	H	6.124	6.008	0.116	6.087	0.037
48	isopropyl	phenyl	H	OH	OCH_3_	6.353	6.283	0.070	6.471	−0.118
49	isopropyl	phenyl	CH_3_	H	H	5.190	5.196	−0.006	5.609	−0.419
50	isopropyl	phenyl	H	H	CH_2_OH	6.389	6.433	−0.044	6.233	0.156
51 *	isopropyl	phenyl	H	H	(CH_2_)_2_OH	6.991	6.572	0.419	6.431	0.560
52 *	isopropyl	phenyl	H	H	(CH_2_)_3_OH	7.009	6.848	0.162	6.975	0.034
53	isopropyl	phenyl	H	H	O(CH_2_)_2_OH	7.022	7.031	−0.009	6.905	0.117
54 *	isopropyl	phenyl	H	H	O(CH_2_)_3_ OH	7.237	6.979	0.258	6.859	0.378
55 *	isopropyl	phenyl	H	H	OCH (CH_2_OH)_2_	5.064	5.607	−0.543	5.438	−0.374
56	isopropyl	phenyl	H	O(CH_2_)_2_OH	H	5.850	5.762	0.088	5.815	0.035
57	isopropyl	phenyl	H	H	O(CH_2_)_3_N (CH_3_)_2_	6.465	6.512	−0.047	6.535	−0.070
58	n-propyl	2-methylphenyl	H	Cl	OH	7.721	7.538	0.183	7.279	0.442
59	n-propyl	2-methylphenyl	H	Cl	O(CH_2_)_2_ OH	7.959	7.933	0.026	8.154	−0.195
60	isopropyl	2-methylphenyl	H	Cl	(R)-OCH_2_ CH(CH_3_) CH_2_OH	8.041	8.026	0.015	8.031	0.010
61	isopropyl	2-methylphenyl	H	Cl	(S)-OCH_2_ CH(CH_3_) CH_2_OH	8.013	8.061	−0.048	7.907	0.106

* Test set.
